# Decision tree-based machine learning algorithm for prediction of acute radiation esophagitis

**DOI:** 10.1016/j.bbrep.2025.101991

**Published:** 2025-03-28

**Authors:** Mostafa Alizade-Harakiyan, Amin Khodaei, Ali Yousefi, Hamed Zamani, Asghar Mesbahi

**Affiliations:** a Department of Radiation Oncology, Faculty of Medicine, Tabriz University of Medical Sciences, Tabriz, Iran; b Faculty of Electrical and Computer Engineering, University of Tabriz, Tabriz, Iran; c Department of Medicinal Chemistry, School of Pharmacy, Tabriz University of Medical Sciences, Tabriz, Iran; d Medical Physics Department, Faculty of Medicine, Tabriz University of Medical Sciences, Tabriz, Iran; e Molecular Medicine Research Center, Tabriz University of Medical Sciences, Tabriz, Iran

**Keywords:** Radiation-induced esophagitis, Decision tree classifier, Predictive modeling, Machine learning, Radiotherapy, Treatment planning

## Abstract

**Background:**

Radiation-induced esophagitis remains a significant challenge in thoracic and neck cancer treatment, impacting patient quality of life and potentially limiting therapeutic efficacy. This study aimed to develop and validate a decision tree-based model for predicting acute esophagitis grades in patients undergoing chemoradiotherapy.

**Methods:**

Data from 100 patients receiving thoracic and neck radiotherapy were analyzed. The dataset comprised 33 features, including demographic, clinical, and dosimetric parameters. A decision tree classifier was implemented for both binary (Grade ≥2 vs. <2) and multi-class (Grades 1, 2, and 3) classification. Model performance was evaluated using standard metrics including accuracy, precision, recall, and F1-score.

**Results:**

The binary classification model achieved 97 % accuracy in distinguishing acute esophagitis. The multi-class model demonstrated 98 % accuracy in predicting specific grades. Key predictive features included V40 (volume receiving 40 Gy), V60, and average esophageal dose. The model generated interpretable decision rules, with V60 ≥ 2.3 strongly indicating Grade 3 esophagitis.

**Conclusions:**

The decision tree model demonstrates high accuracy in predicting radiation-induced esophagitis grades while maintaining clinical interpretability. This approach offers potential for treatment optimization and personalized risk assessment in radiotherapy planning. The model's transparency and reliability make it a promising tool for clinical decision support in radiation oncology.

## Highlights

• Development and Validation of a Decision Tree Model for Predicting Acute Radiation Esophagitis Grades in Thoracic and Neck Cancer Patients.
• Prediction of acute esophagitis with machine learning algorithms.
• Use clinical, pathological and physical data for prediction of esophagitis.

## 1 Introduction

Cancer is the issue that is the most difficult to treat for current medicine, which is the main responsible for the millions of deaths that take place annually worldwide [1,2]. Its management usually involves different methods such as surgery, chemotherapy, and radiotherapy [3]. Radiotherapy is an example of this service that has allowed it to be one of the key treatments for cancer because it is capable of targeting precisely the tumor cells and avoiding the surrounding normal tissues [4,5]. However, as with any other therapy, radiotherapy has its own risks. The treatment of thoracic and neck tumors include the esophagus, a vital organ often in close proximity to many tumors in these areas, being irradiated frequently [6,7]. This is a symptom that might occur due to exposure to radiation that is called acute radiation esophagitis and it is a painful and disabling side effect that has major effects on patients' quality of life. Among the causes of radiation-induced esophagitis being severe, the radiation dose, the volume of the esophagus exposed, and the patient's clinical condition should be mentioned too [8,9].

These issues are generally graded based on the existing reference guideline such as the Radiation Therapy Oncology Group (RTOG) classification which intensifies the evaluation and management of side effects with an organized approach. Yet, the fact that patients have different responses to the treatment requires the use of more personalized techniques to anticipate and eliminate such unwanted consequences [10,11]. Developments in radiotherapy practices like intensity-modulated radiotherapy (IMRT) and proton therapy have been the breakthrough in the cancer treatment by improving the precision of the radiation that is delivered. These methods are not only about the enhancement of tumor control but also the minimization of the beating up the adjacent tissues. However, even with the technological advances hitherto achieved, radiation-induced esophagitis is still a common and challenging issue, highlighting the need for new predictive models. The use of artificial intelligence AI has brought with it a new dimension to healthcare which has been greatly seen in the field of oncology. Integration of artificial intelligence (ML) algorithms into clinical workflows has facilitated the prediction of treatment outcomes and side effects with precision [12,13].

The traditional dose-volume histogram and the radiobiological model are not the only ways that modern machine learning (ML) models are different. These last two are the range of their applicability. ML models can process extensive data that may embrace demographic, clinical, and dosimetric details, something that most traditional methods can't do which is their reliance on a few parameters only [14]. This is where the difference lies. For example, through the capability, even the riskiest factors could be predicted more precisely for every patient. This was based on the combination of if you or your friend are the author of the research above words into a single phrase you can present as the end product. The intelligent method used in backend processes of the decision tree classifier which part of the AI techniques is allowed the successful management of the application. All the features were selected and were transformed into 1 or 0 and then went in the input node. The main purpose of the models is to separate the data into different groups, each with different labels [15]. In this sentence, it is a classifier that sequentially divides into groups by features' thresholds and finally assigns it to a patient's class. This type of structure is not only perfectly predicting but also the doctors who use these models can deeply understand them and justify the model's decision. This technique is more likely to increase the trust of the clinicians who are to use the model in the clinics, which is an essential part of the process of adoption of the machine learning model. Yet, there is a notable gap in this promising field, specifically, the exact prediction of acute esophagitis grade influenced by chemoradiotherapy. Closely related to this issue a study on How Screen Time Is Rewiring Your Kids Brain and how to prevent it from happening was published, in the further reading you can get specific information about why the problem exists. However, it is a massive problem due to lack of proper diagnostic tools [16].

A problem of prediction persisted in most of the published and so far existing research work, e.g. studies on esophagitis are mainly designed for classification - either yes/no type or intermediate grades. To close this gap, the study of grade diagnosis by the decision tree algorithm is proposed to predict the exact grade of esophagitis. Therefore, a tree query first gets the study's data and then divides it according to the features. Then the final result is the predicted class of the patient. Data from the study demonstrated that it was possible to incorporate features from various sources, the survey, and the registry through the decision tree [17].

Regarding the analysis of acute radiation esophagitis data, recent research has also been conducted, among which is research number [18]. Certainly, in some of these studies, such as [19], artificial intelligence and machine learning approaches have been used for data analysis. Similar research has also been conducted using artificial intelligence approaches, including [20] studies focused on renal cancer data. Among other research conducted using machine learning or deep learning methods [21], can be mentioned. Recent research has also been conducted in the field of radiation oncology regarding the application of artificial intelligence [22].

This study aims to show that decision tree models combined with large datasets can very well predict radiation-induced esophagitis severity. Radiation Esophagitis Severity Standardizing this idea could greatly heighten the quality of patient care by enabling clinicians to identify at-risk individuals in the initial phases and adapt treatment plans accordingly.

## 2 Materials and methods

### 2.1 Data collection and dataset description

This research sets out to forecast radiation induced esophagitis grades by using the dimensional machine learning system. Availability of data from the 100 patients to whom were concurrently receiving radiotherapy and chemotherapy for the thoracic and neck tumor [23,24]. The dataset was collected from two independent hospitals in Tabriz, Iran, each following standard national oncology guidelines for radiotherapy and chemotherapy. To ensure consistency in data quality and treatment protocols, we verified that both hospitals adhered to similar dose fractionation schedules and clinical management strategies. However, slight variations in patient demographics and institutional practices may exist, which could influence model performance in different settings. Addressing this limitation, we recommend future studies include multi-center datasets spanning diverse geographic locations to enhance model generalizability. There is a variety of parameters mentioned for the look of the patient. Demography of the patient (eg., sex, age), clinical details (eg., tumor stage, comorbidities), and dosimetric data (eg., maximum and average esophageal doses) belong to the set of 33 features [25]. There was no error in the data collection process that involved carrying out a careful stigmatization of the data along with a simplistic analysis to clear any issues of predictive significance. This allowed the decision-making to be more personalized and accurate and provided insights that enable more reliable clinical interpretation of the results. Every characteristic of the dataset has been mentally examined before making radiation-induced esophagitis a connection to it. The demographic variables offered a base level for the patient-specific susceptibilities so that all the differences were identified while the dosimetric metrics visually presented the relationship between treatment intensity and adverse effects. The data proved to be an epitome of an ideal dataset which consequently was very indispensable and responsible for the creation of the resilient forecasting model that could work with different key features of the patients.

### 2.2 Methodology

**Fig. 1:**
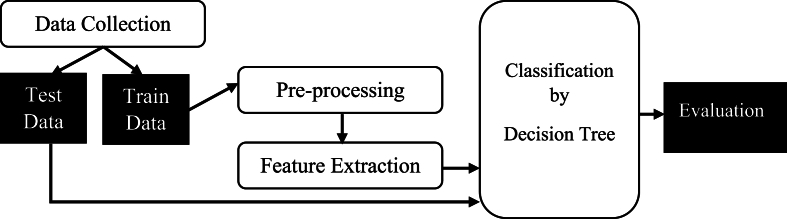
Workflow of the Proposed Methodology (A diagram detailing the steps: data collection, preprocessing, feature selection, model training, evaluation, and interpretation.).

The methodology employs a systematic approach, detailed in Fig. 1, comprising five distinct stages.

#### 2.2.1 Data preprocessing

Missing values were interpolated, outliers were factored, and numerical features were standardized for there maintenance through the whole dataset. [26]. Also, encoding of categorical variables by one-hot encoding was performed to enable their integration into the model with a minimum possible loss of information [27,28]. To ensure data completeness and reliability, a systematic preprocessing approach was applied. Missing values, constituting 3.5 % of the dataset, were handled using multiple imputation techniques: numerical variables were imputed using k-nearest neighbors (KNN) imputation, while categorical variables were imputed using mode imputation. Additionally, interquartile range (IQR) analysis was employed to identify and manage potential outliers. Feature selection using Recursive Feature Elimination (RFE) helped mitigate the effects of correlated or redundant features, ensuring a stable model. This study utilized a cross-sectional dataset where all patient characteristics and treatment parameters were recorded at a single time point. Consequently, temporal changes in treatment response or patient condition over the course of therapy were not explicitly captured.

#### 2.2.2 Feature selection and extraction

Methods like recursive feature elimination and correlation analysis were employed for finding the most important features thereby keeping the dimensionality and the model's performance at the same level. Feature selection was performed using a two-stage process. First, statistical filtering was applied to remove highly correlated features (Pearson's correlation coefficient >0.85) and those with minimal variance. Then, Recursive Feature Elimination (RFE) was used to identify the 33 most predictive features from an initial set of 42. This approach helped maintain model accuracy while preventing overfitting.To be short, the aim of this step was to wipe out those parameters which will not play a major role in predicting the target Class. To ensure the stability and reliability of the model despite the relatively small sample size, we applied rigorous cross-validation techniques, including K-Fold Cross Validation (k = 10) and Leave-One-Out Cross Validation (LOOCV). These techniques help mitigate overfitting and improve the generalizability of the model. Additionally, feature selection was performed using Recursive Feature Elimination (RFE) to ensure that only the most relevant features contributed to the final predictive model.

#### 2.2.3 Model training and testing

The use of a decision tree was the result of its multifunctional characteristic which gives the opportunity to solve both multi-class classification tasks and provide understandable results. In Fig. 1, decision trees are mathematical models that identify the right threshold of features and make the splits to produce trees with nodes and branches that become leaves (end of the last branch) which the classes have been as predicted. This model is a good fit for the data provided in this case as numerical and categorical features can be handled in one model in an effective manner. The decision tree was trained in a supervised learning approach [29].

Classifiers can be train and test by several validation strategies such as K-fold, leave one out, hold-out, and resubstitution. In hold-out strategy, the dataset was partitioned into training and testing sets by percentage (like 70-30 %). The model changed its internal weights to correct classification errors during training using training data. The tree depth and the minimum sample size per leaf are the most important hyperparameters and the accuracy and generalizability should be simultaneously balanced. The k-fold method divides the dataset into k folds (k value is 10). The model is trained on 9 folds and tested on the 1 remaining fold, and this process is repeated for each fold. Leave one out is a special version of k-fold. Resubstitution use the train set as test set and give an optimistic estimate of model performance. To assess the model's generalizability despite the absence of external validation, we employed robust internal validation techniques. 10-fold Cross-Validation (10-CV) and Leave-One-Out Cross Validation (LOOCV) were utilized to evaluate model performance across different partitions of the dataset. Additionally, a sensitivity analysis was conducted to ensure the model's robustness across various patient subgroups. To prevent overfitting and improve generalizability, several regularization techniques were applied. Cost-complexity pruning (CCP) was implemented to simplify the decision tree by eliminating branches with minimal contribution to overall accuracy. Additionally, 10-fold Cross-Validation (10-CV) and Leave-One-Out Cross Validation (LOOCV) were utilized to ensure model stability across different subsets of the data. The final model was evaluated on an independent test set (30 % of the dataset), achieving 97 % accuracy, confirming its ability to generalize effectively beyond the training data. To maintain interpretability and clinical applicability, cost-complexity pruning (CCP) was applied to simplify the decision tree while preserving its predictive power. Recursive Feature Elimination (RFE) was also utilized to refine feature selection, ensuring that only the most critical 33 features were retained. These techniques enhanced the model's transparency and usability for healthcare professionals.

The performance of the decision tree model was assessed with standard metrics in classification tasks, including accuracy, precision, recall, and F1-score. In the binary classification case, (e.g., Grade≥2 vs. Grade <2), confusion matrices were designed to estimate the proportions of true positive (TP), true negative (TN), false positive (FP), and false negative (FN). In multi-class classification, a more intricate confusion matrix was used to explain the distribution of predictions among the three esophagitis grades (1, 2, and 3). The proportion of instances correctly predicted was how accuracy was computed while precision and recall added information on the reliability and sensitivity of the model. Confusion matrices for binary and multi-class classification problems were made. Metrics like accuracy, precision, recall, and F1-score were found for each class, thus, the evaluation of the model's overall effectiveness was obtained.

#### 2.2.4 Interpretation and visualization

Rules and decision boundaries that had been found were translated into ideas for use as well as the transparency of the model's predictions. Visualization such as confusion matrices and feature importance plots were produced with the aim to clarify those interpretations.

Table Patient demographic, clinical, and dosimetric features used in the predictive model e 1.

### 2.3 Decision tree classifier

**Fig. 2:**
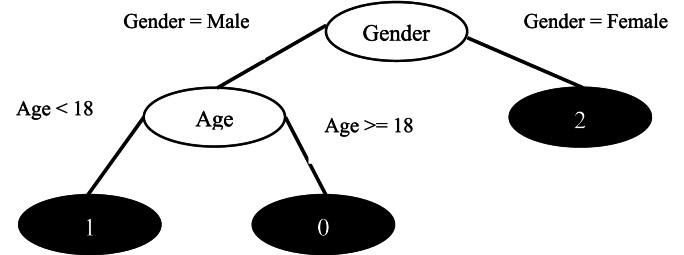
Decision Tree Classifier (A tree diagram showing nodes for features and thresholds, leading to leaf nodes representing predicted classes.).

The decision tree classifier was employed for both binary and multi-class problems. The plot which was included in Fig. 2 depicts this model, which is simply a partition of the dataset based on feature thresholds, producing an understandable set of rules to be followed by predictions of the model [30,31].

#### 2.3.1 Binary classification

A class of patients were split into two groups, mild esophagitis (Grade <2) and server esophagitis (Grade ≥2). Which node that is the deciding node rule was fired that proved to be useful in the classification process for example in the case of V40 (the volume receiving 40 Gy) and the average esophageal dose. To the contrary, the leaf nodes stood in separation symbolizing the classes which were successfully predicted.

#### 2.3.2 Multi-class classification

This classifier was then extended to predict the three grades, namely; 1, 2, and 3. There were additional decision thresholds introduced which increased the complexity of the tree although its interpretability remained unchanged. The formulation rules from the tree provided a clear pathway of the insights how a patient's characteristic is in relation to the esophagitis grades/kinds**.** For example, 3 rules can be extracted from Fig. 2 decision tree.

### 2.4 Statistical analysis and model performance

The performance of the model was evaluated using both binary and multi-class metrics. In machine learning, various established metrics are employed to assess model performance. Classification tasks are generally divided into two categories: binary-class and multi-class [33]. The primary goal is to distinguish one specific class from others. In this study, the objective was to identify samples with a grade of ≥2. In binary classification, four possible outcomes are defined: True Positive (TP): Cases where the sample truly belongs to Grade ≥2, and the model correctly predicts Grade ≥2. False Positive (FP): Cases where the sample belongs to Grade <2, but the model incorrectly predicts Grade ≥2. True Negative (TN): Cases where the sample belongs to Grade <2, and the model correctly predicts Grade <2. False Negative (FN): Cases where the sample belongs to Grade ≥2, but the model incorrectly predicts Grade <2. These outcomes form the foundation for various performance metrics commonly used in binary classification. Among them, accuracy is one of the most widely recognized metrics. It is calculated using the following formula:

(1) $$\mathrm{Accuracy}=\frac{TP+TN}{TP+TN+FP+FN}$$

**Fig. 3:**
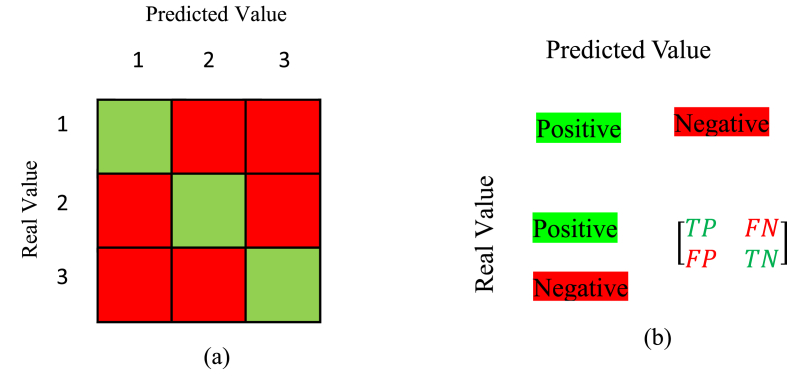
Confusion Matrix (A matrix illustrating actual vs. predicted values for both binary and multi-class problems.).

The study also defines a multi-class classification problem, where the goal is to design a model that can accurately predict the grade based on the features of the samples. In this case, similar metrics can be defined, but the number of possible outcomes depends on the number of classes. The study defines three grades, resulting in 9 possible outcomes, which necessitate using a confusion matrix for performance analysis, as shown in Fig. 3. In the binary classification scenario shown in part (a) of Fig. 3, the four outcomes are displayed in a matrix. In part (b), the confusion matrix for a 3-class classification problem is presented, where the number of rows and columns represents the actual and predicted classes, respectively. The diagonal elements of the matrix are the correctly predicted samples for each class. For evaluating other metrics such as accuracy, all elements of the matrix are considered. The (2) formula for accuracy in multi-class classification is:

(2) $$\mathrm{Accuracy}=\frac{\sum_{i=1}^{n}C\left(i,i\right)}{\sum_{i=1}^{n}\sum_{j=1}^{n}C\left(i,j\right)}$$

Where i and j refer to the row and column indices, and all elements of the matrix are summed in the denominator, with the diagonal elements considered in the numerator. The multi-class model demonstrated similar success, achieving 98 % accuracy. Confusion matrices (Fig. 3) provided insights into classification trends and errors. High diagonal values in the confusion matrix confirmed the model's reliability in distinguishing between grades [34,35].

Fig. 3 presents the confusion matrix, which illustrates the alignment of the model's predictions with actual patient outcomes. The high rates of true positives (TP) and true negatives (TN) reflect the model's robustness, while the low occurrences of false positives (FP) and false negatives (FN) emphasize its practical applicability in clinical settings. These performance metrics are especially crucial in healthcare, where misclassification can have serious consequences, potentially compromising patient safety and treatment efficacy. From the confusion matrix, other components for evaluating the performance of machine learning models, such as recall, precision, sensitivity, and specificity, can also be calculated.

## 3 Results

Multiple experiments have been conducted based on the concepts of the proposed algorithmic approach, which are discussed further below. It is important to note that these experiments were carried out in the MATLAB 2018 software environment.

### 3.1 Binary classification performance

**Fig. 4:**
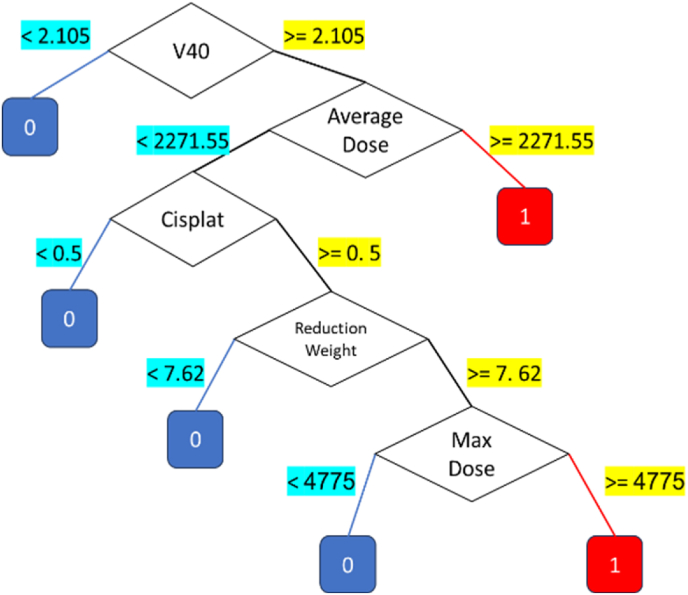
Decision tree for binary-class classification.

One of the goals of this research is to provide an interpretable intelligent model that can predict the grade based on the features collected from the study samples. The defined binary classification task by decision tree classifier is shown on Fig. 4, which categorized patients into mild (Grade <2) and severe (Grade ≥2) esophagitis, yielded highly impressive results. The decision tree model achieved an outstanding accuracy of 97 %, showcasing its precision in effectively differentiating between the two categories.To further enhance interpretability, we have provided a decision tree diagram (Fig. 4), illustrating the key decision paths utilized by the model. This visualization allows clinicians to understand how predictive features contribute to classification outcomes, facilitating real-world implementation.The model's reliability was further reinforced by its high precision and recall values, indicating a minimal margin of error in its predictions.

**Fig. 5:**
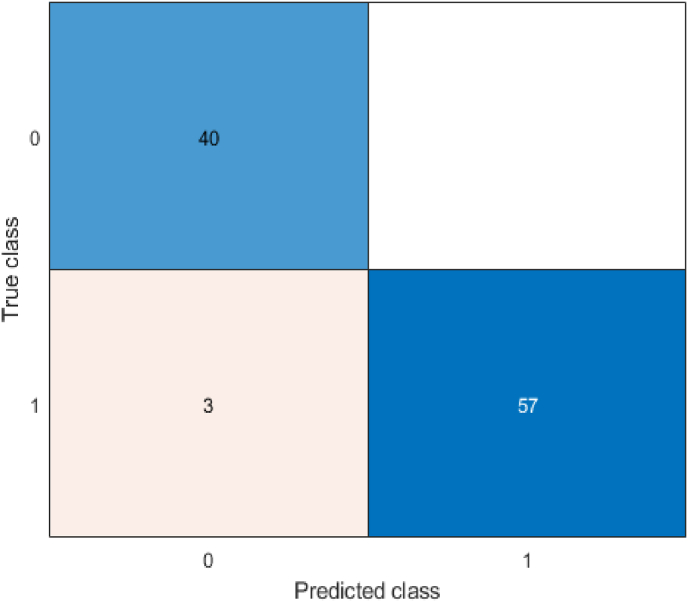
Confusion matrix for binary-class classification.

Feature importance analysis identified that dosimetric parameters such as V40 (volume receiving 40 Gy) and the average esophageal dose were pivotal in the classification process. These features align with established clinical knowledge, supporting the model's credibility. The mentioned decision tree classifier confusion matrix is shown on Fig. 5.

Fig. 5 and the corresponding matrix indicate the good performance of the decision tree model in accurately predicting and classifying the sample types, with a low error rate.

**Fig. 6:**
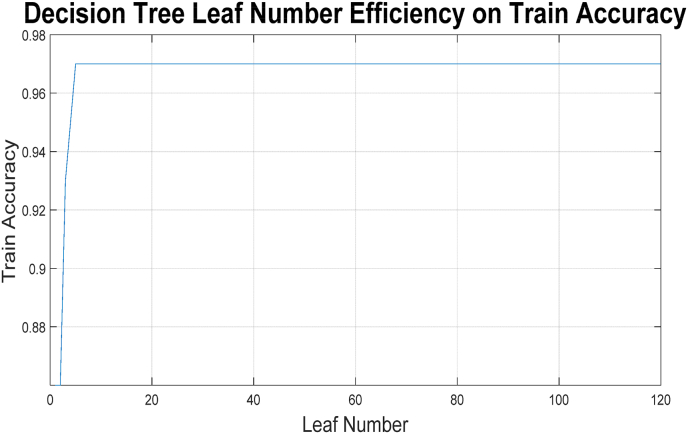
Leaves efficiency on the obtained results of binary class decision tree.

To construct this decision tree and determine how deep it should go and how many leaves it should have, tree pruning techniques can be utilized. The 10-fold strategy can also be employed to compare the average accuracy results of the training and testing phases. To this end, an experiment has been designed to examine the impact of increasing the number of leaves in the tree on the accuracy achieved with the aforementioned strategy. The results are illustrated in Fig. 6, where the horizontal and vertical axes correspond to the number of leaves and the average accuracy obtained, respectively.

**Fig. 7:**
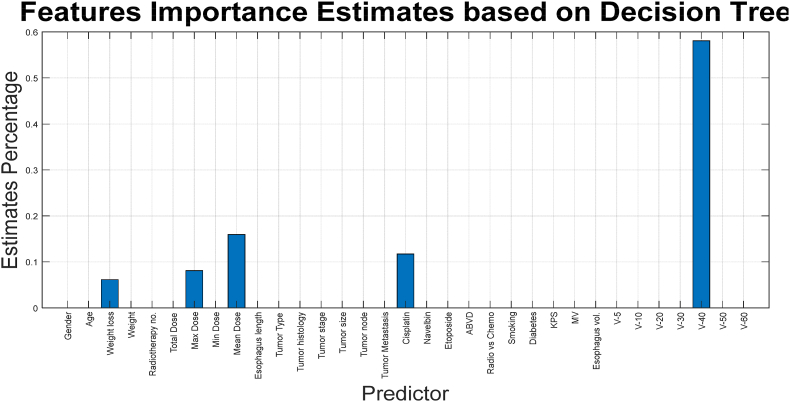
Weighting features based on the designed decision tree for binary class classification.

In Fig. 6, it is evident that after a certain number of leaves, no changes in the results are observed. This may indicate that some features do not influence the accuracy of the decision tree learning model. Based on this decision tree, the importance of various features can also be weighted in a certain way. Fig. 7 illustrated the importance of each feature based on the designed decision tree, where the horizontal and vertical axes correspond to the feature names and the estimated percentage, respectively.

As shown in Fig. 7, the value and importance of the features that are positioned higher up and closer to the root of the decision tree are also greater (see Table 1).

**Table 1:** Features extracted from patient data.

Values	Type	Name	Number
0/1	Binary	Gender	1
[20–70]	Numeric	Age	2
[[4], [5], [6], [7], [8], [9], [10], [11], [12], [13], [14], [15], [16], [17], [18]]	Percentage	Weight reduction after treatment	3
[18–115]	Numeric	Weight before treatment	4
[[10], [11], [12], [13], [14], [15], [16], [17], [18], [19], [20], [21], [22], [23], [24], [25], [26], [27], [28], [29], [30], [31], [32], [33], [34], [35], [36], [37], [38], [39], [40]]	Numeric	Radiotherapy sessions	5
[1980–7200]	Numeric	Prescribed dose to tumor	6
[3075–8900]	Numeric	Maximum dose to esophagus	7
[50–3050]	Numeric	Minimum dose to esophagus	8
[329–4660]	Numeric	Average dose to esophagus	9
[[3], [4], [5], [6], [7], [8], [9], [10], [11], [12], [13], [14], [15], [16], [17], [18], [19], [20], [21], [22], [23], [24], [25]]	Numeric	Esophagus length in field	10
{Larynx, Mouth, Lung, Nasopharynx, Lymphoma, Spine}	Categorical	Tumor location	11
{SCC, SCLC, NSCLC, Metastatic spinal tumor, Hodgkin lymphoma}	Categorical	Tumor histology	12
[[1], [2], [3], [4], [5], [6]]	Numeric	Tumor stage	13
[0–9]	Numeric	T	14
[0–9]	Numeric	N	15
[0–9]	Numeric	M	16
0/1	Binary	Cisplat!	17
0/1	Binary	Navelb!	18
0/1	Binary	Etoposide	19
0/1	Binary	ABVD	20
0/1	Binary	Radiotherapy only or combined with chemotherapy	21
0/1	Binary	Smoker	22
0/1	Binary	Diabetic	23
[60–100]	Numeric	KPS	24
[[6], [7], [8], [9], [10], [11], [12], [13], [14], [15], [16], [17], [18]]	Numeric	Energy used for tumor treatment	25
[6–111]	Numeric	Volume of esophagus in field	26
[2–103]	Numeric	V5	27
[2–103]	Numeric	V10	28
[0-99]	Numeric	V20	29
[0-99]	Numeric	V30	30
[0-95]	Numeric	V40	31
[0-46]	Numeric	V50	32
[0-31]	Numeric	V60	33

### 3.2 Rule extraction and interpretability of binary class classification

One of the most significant advantages of the decision tree model is its interpretability. The rules derived from the model provide a transparent mechanism for understanding how specific features influence the prediction of esophagitis grades. These rules, summarized in Table 2, offer clear insights into the decision-making process. For example: Rule 1 If V60 ≥ 2.3, then the predicted grade is 3. Rule 2 If V60 < 2.3 and V40 ≥ 2.105 and average dose ≥2271.55, then the predicted grade is 2.

**Table 2:** Extracted rules from binary-class decision tree.

Rule	Condition	Predicted Grade
1	(V40≥2.105) AND (Average Dose≥2271.55)	2 or 3
2	(V40≥2.105) AND (Average Dose <2271.55) AND (Cisplat = = 1) AND (Reduction weight≥7.62) AND (Maximum Dose≥4775)	2 or 3
3	(V40≥2.105) AND (Average Dose <2271.55) AND (Cisplat = = 1) AND (Reduction weight≥7.62) AND (Maximum Dose <4775)	1
4	(V40≥2.105) AND (Average Dose <2271.55) AND (Cisplat = = 1) AND (Reduction weight <7.62)	1
5	(V40≥2.105) AND (Average Dose <2271.55) AND (Cisplat = = 0)	1

These rules closely correspond with clinical expectations, reinforcing the decision tree model's validity as a tool for decision support in clinical settings.

### 3.3 Multi-class classification performance

**Fig. 8:**
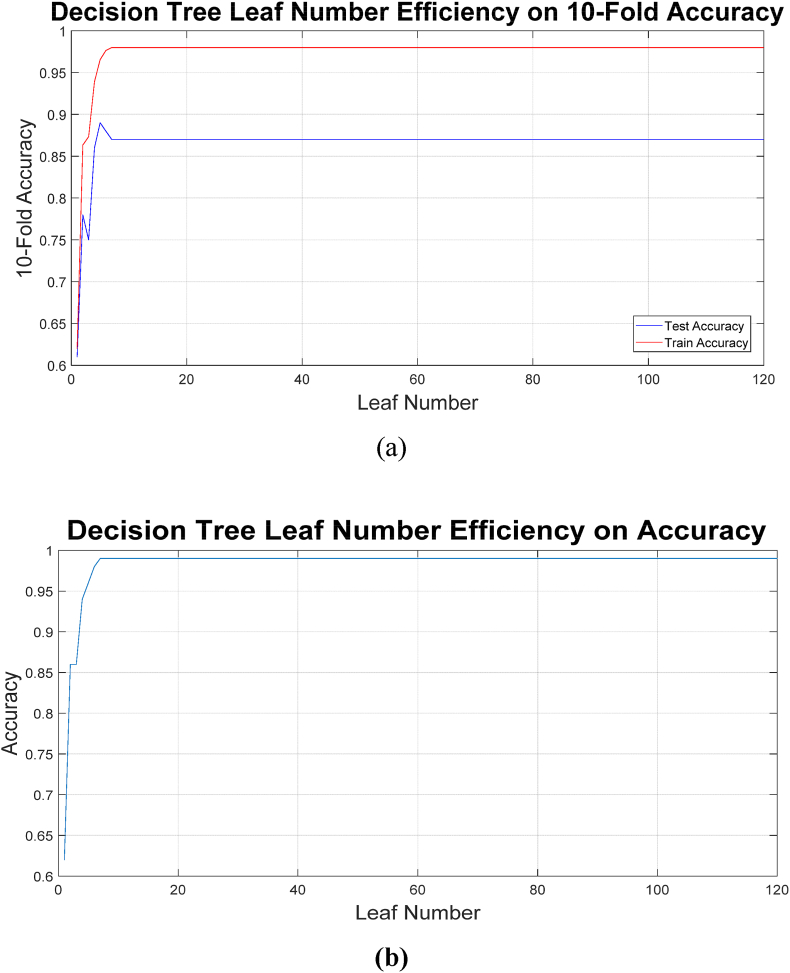
Leaves efficiency on the obtained results of (a) 10-fold (b) resubstitution validation.

The multi-class classification task, designed to predict specific grades of esophagitis (1, 2, or 3), introduced a higher level of complexity compared to the binary task. It should be noted that one of the influential parameters in decision trees is the number of leaves and levels. Determining this value affects the balance between training and testing the model and the challenge of overfitting. To this end, trees with a number of leaves ranging from 1 to 120 were trained and tested, and the results are illustrated in Fig. 8 in terms of the number of leaves and the resulting accuracy. Section (a) shows the results obtained from the 10-fold technique, while Section (b) presents the results obtained from the resubstitution technique.

In the figure related to k-fold, there are two graphs: one corresponds to the training phase and the other to the testing phase. It is natural for the results of the training phase to be better than those of the testing phase; however, increasing the number of leaves in the tree, which also increases the height of the tree, has not had an effect beyond a certain point. Therefore, pruning the tree can lead to an optimized tree.

**Fig. 9:**
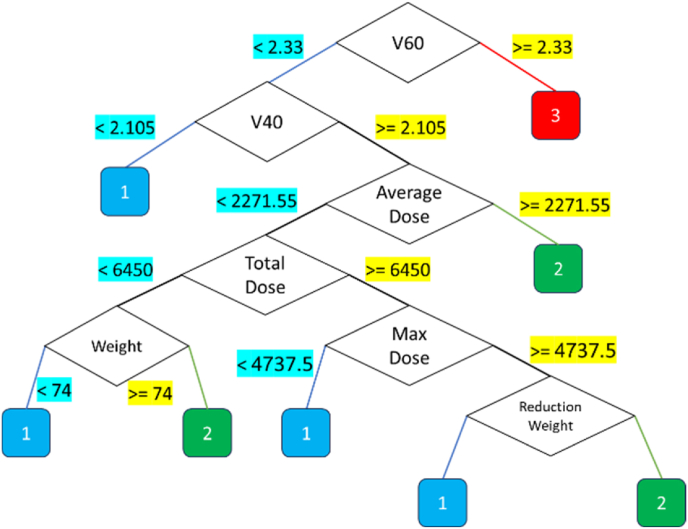
Decision tree for multi-class classification.

Despite this, the decision tree model demonstrated exceptional performance, achieving an accuracy of 98 %. This result highlights the model's ability to handle the additional challenges posed by multi-class prediction effectively. By considering the pruning of the decision tree, obtained decision tree structures used for multi-class classification is illustrated on Fig. 9. These visualizations depict the hierarchical decision-making pathways, providing a clear understanding of how specific features and thresholds determine outcomes. As illustrated in Fig. 9, the decision tree model utilizes a structured hierarchical approach for classification. To ensure clarity and interpretability, cost-complexity pruning was applied to remove unnecessary branches, and Recursive Feature Elimination (RFE) was used to refine feature selection. This streamlined decision-making process enhances the transparency of the model, making it more accessible for clinical use.

**Fig. 10:**
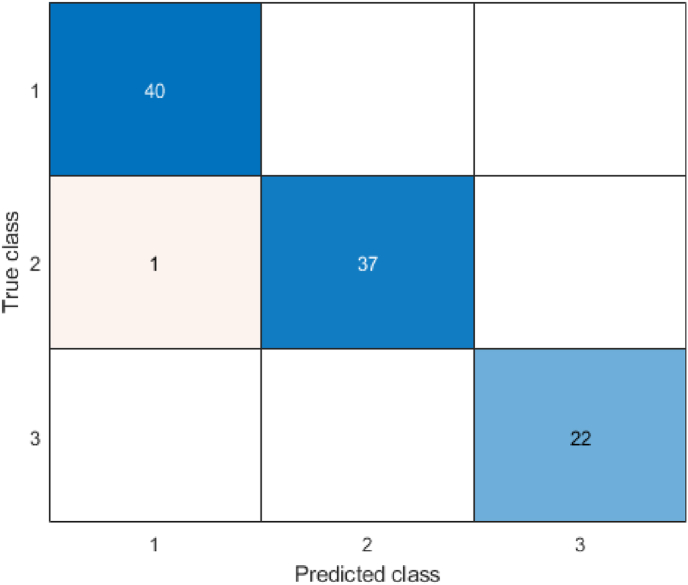
Confusion matrix for multi-class classification.

The confusion matrix resulting from applying this tree to the training of all the data is also illustrated in Fig. 10. Fig. 10 presents the confusion matrix for the multi-class classification, clearly illustrating the alignment between predicted and actual grades. Correct classifications are prominently displayed along the diagonal, while the off-diagonal elements, representing misclassifications, are minimal. Notably, predictions for Grade 3 achieved a flawless accuracy of 100 %. While Grades 1 and 2 showed minor overlaps, these can be attributed to shared characteristics among patients in these categories.

Fig. 10 details the structure of the multi-class decision tree, illustrating how features influence the prediction of Grades 1, 2, and 3.

### 3.4 Rule extraction and interpretability of multi-class classification tree

**Fig. 11:**
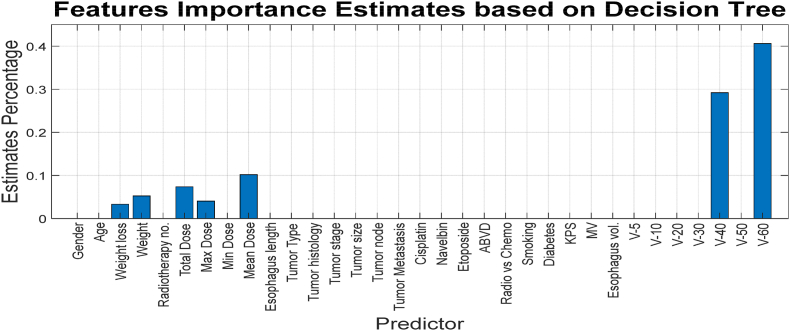
Weighting features based on the designed decision tree for for Multi-Class Classification.

Using the mentioned tree can be weight the value of each feature, as shown in Fig. 11.

As shown in Fig. 11, each of the features has a specific value in the designed decision tree. However, some features have a weight of zero, which is due to the constraints on tree growth and its pruning, and this is logical from the perspective of feature selection as well. In other words, this chart has weighted the features listed in Table 3. Fig. 9 results rules are written in Table 3.

**Table 3:** Extracted rules from multi-class decision tree.

No.	Input	Output
1	(V60≥2.3)	3
2	(V60 < 2.3) AND (V40≥2.105) AND (Average Dose≥2271.55)	2
3	(V60 < 2.3) AND (V40≥2.105) AND (Average Dose <2271.55) AND (Total Dose <6450) AND (Weight≥74)	2
4	(V60 < 2.3) AND (V40≥2.105) AND (Average Dose <2271.55) AND (Total Dose≥6450) AND (Maximum Dose >4737) AND (Reduction weight≥7.62)	2
5	(V60 < 2.3) AND (V40≥2.105) AND (Average Dose <2271.55) AND (Total Dose≥6450) AND (Maximum Dose >4737) AND (Reduction weight <7.62)	1
6	(V60 < 2.3) AND (V40≥2.105) AND (Average Dose <2271.55) AND (Total Dose≥6450) AND (Maximum Dose≤4737)	1
7	(V60 < 2.3) AND (V40≥2.105) AND (Average Dose <2271.55) AND (Total Dose <6450) AND (Weight <74)	1
8	(V60 < 2.3) AND (V40 < 2.105)	1

As indicated in Tables 3 and it is possible to predict the output using only 7 features, which were also discussed in the previous figure results.

## 4 Discussion

The findings of this study underscore the significant potential of machine learning, particularly decision tree models, in enhancing clinical decision-making for predicting treatment-related complications. By leveraging patient-specific demographic and dosimetric data, this research has achieved high predictive accuracy while providing a transparent and interpretable framework that aligns with clinical needs.

### 4.1 Interpretability and clinical relevance

One of the key strengths of the decision tree model lies in its interpretability, which sets it apart from many "black-box" machine learning methods. In clinical practice, interpretability is not merely a desirable feature—it is a necessity. Healthcare professionals must understand the reasoning behind predictions to trust and incorporate them into patient care. Decision trees excel in this area by offering clear, actionable rules that describe relationships between variables, such as the impact of **V60**, **V40**, and esophagitis grades. This makes them particularly well-suited for integration into routine workflows. Notably, the model's findings reinforce established clinical hypotheses while uncovering new patterns that could refine treatment strategies. For example, the critical role of dosimetric parameters in predicting esophagitis severity was evident. Thresholds for **V60** and **V40** emerged as particularly significant, suggesting that careful optimization of radiation dosing can substantially reduce adverse effects. These insights can directly influence clinical protocols and enhance patient outcomes, underscoring the decision tree's value as a reliable decision-support tool. Ensuring that predictive models are interpretable is essential for clinical adoption. By simplifying the decision tree structure and visualizing key decision paths, we have improved the model's transparency and potential for real-world application. Future studies should explore integrating decision-support tools that translate these predictions into actionable recommendations for clinicians. The predictive capability of this model provides a valuable tool for early risk stratification in radiation oncology. Compared to conventional clinical assessment methods, which rely on empirical dose-volume constraints and physician experience, the decision tree model offers a data-driven, individualized risk prediction approach. This can aid in optimizing treatment planning by enabling dose modifications for high-risk patients and implementing proactive supportive care strategies such as pharmacologic interventions and nutritional counseling. Future work should focus on integrating this model into radiation therapy decision-support systems to further validate its impact on clinical decision-making and patient outcomes.

### 4.2 Comparative strengths of decision tree models

The decision tree model stands out for its unique combination of accuracy, simplicity, and interpretability, making it an ideal choice for clinical applications. While alternative machine learning techniques, such as neural networks or ensemble models, might achieve slightly higher accuracy, their lack of transparency often limits their utility in healthcare, where interpretability is paramount. In this study, the decision tree model demonstrated high precision and recall across both binary and multi-class classification tasks, showcasing its robustness. Additionally, feature importance analysis highlighted its ability to focus on the most relevant parameters—such as dosimetric data and demographic factors—while minimizing distractions from irrelevant noise. This efficiency reduces computational overhead, aligning with the practical constraints of clinical environments, where time and resources are often limited [38,39].

### 4.3 Implications for personalized medicine

The high accuracy of the model in predicting acute esophagitis grades holds significant promise for advancing personalized medicine. Tools like the decision tree model empower clinicians to identify high-risk patients proactively and customize treatment plans tailored to individual needs. For instance, patients with dosimetric profiles indicating a high risk of severe esophagitis could benefit from adjusted radiation doses, alternative therapies, or enhanced supportive care during treatment. Integrating such predictive models into clinical workflows streamlines decision-making, reduces the need for trial-and-error treatment adjustments, and ultimately improves patient outcomes. By bridging advanced analytics with patient-centered care, these models pave the way for a more efficient and effective healthcare system.

### 4.4 Comparative analysis with previous studies

When compared to previous research, the decision tree model demonstrates clear advantages. While machine learning techniques like support vector machines (SVMs) and neural networks often provide comparable accuracy, their lack of interpretability makes them less appealing for clinical use. Clinicians are more likely to adopt models that offer transparent and understandable explanations for their predictions, making the decision tree approach particularly well-suited for healthcare applications [40,41].

### 4.5 Limitations and future directions

Despite its promising results, this study has several limitations that warrant further exploration. The relatively small dataset of 100 patients may limit the generalizability of the findings. One of the primary limitations of this study is the relatively small sample size (n = 100), which may impact the generalizability of the findings. While our results align with previous studies utilizing similar datasets, we acknowledge the importance of validating the model on larger, multi-institutional datasets [[42], [43], [44], [45], [46], [47]]. Future research should focus on expanding the dataset to include a broader population and assess model performance across different clinical settings. Additionally, prospective external validation should be conducted to further confirm the robustness and applicability of the proposed model. Although our model demonstrated high accuracy, the absence of molecular biomarkers (e.g., inflammatory cytokines, genetic predisposition markers) may limit its predictive power. Future studies should integrate multi-omics data to explore potential biological contributors to acute radiation esophagitis and refine model predictions. Although pruning and cross-validation techniques were applied to minimize overfitting, external validation using completely independent datasets is required to further confirm the robustness of the model. Future studies should evaluate the decision tree model on prospective multi-center datasets to ensure its applicability in diverse clinical settings. Another limitation of this study is that, although data were obtained from two independent hospitals, the findings may still be influenced by regional treatment practices and patient characteristics. Therefore, additional external validation using multi-institutional datasets from different geographic regions is necessary to confirm the broader applicability of the model. Despite rigorous preprocessing, data quality remains an important consideration. While missing values were minimal and handled systematically, the dataset was retrospectively collected, which may introduce biases inherent to observational studies. Future studies should aim to incorporate prospectively collected, high-resolution datasets to further enhance predictive reliability. Although rigorous internal validation techniques were applied, external validation on independent datasets remains essential. Future studies should focus on testing the model's performance in multi-center datasets with diverse patient populations to confirm its generalizability. Another limitation of this study is the use of cross-sectional data, which does not account for temporal variations in patient responses to radiation therapy. Future studies should incorporate longitudinal datasets that track changes in treatment response and esophagitis severity over time. The integration of sequential patient data could enable the development of dynamic predictive models for more precise risk stratification. While decision trees provide unmatched interpretability, exploring complementary machine learning approaches—such as random forests or gradient boosting—could enhance predictive performance. These ensemble methods might capture more complex feature interactions, thereby improving accuracy and reliability.

The lack of longitudinal data restricts the analysis to static predictions. Incorporating dynamic patient monitoring and time-series data in future research could enable the development of adaptive models that adjust predictions in real-time, offering even greater personalization. This study primarily focuses on dosimetric and demographic features. Expanding the model to include biological markers—such as inflammatory cytokines or genetic predispositions—could enrich the feature set and improve predictive capabilities. This would not only enhance accuracy but also provide a more comprehensive understanding of the factors contributing to esophagitis severity. While decision trees excel in interpretability, combining them with advanced algorithms like neural networks or leveraging explainable AI techniques could achieve the best of both worlds—maintaining transparency while enhancing predictive power.

## 5 Conclusion

This study underscores the transformative potential of decision tree models in predicting acute esophagitis grades in patients undergoing radiotherapy and chemotherapy for thoracic and neck tumors. By utilizing patient-specific demographic and dosimetric data, the research demonstrates that decision tree models not only deliver high predictive accuracy but also offer unmatched interpretability, making them ideal for clinical applications. The ability of these models to generate clear, actionable rules bridges the gap between advanced machine learning techniques and practical clinical needs. Key findings, such as the significant role of dosimetric parameters like V60 and V40 in determining esophagitis severity, highlight the clinical relevance of this approach. These insights empower healthcare professionals to tailor treatment plans more effectively, minimizing side effects and improving therapeutic outcomes. Despite its promising results, this study is not without limitations. The relatively small sample size and the lack of longitudinal data present opportunities for further investigation. Future research should focus on validating the model with larger, more diverse datasets and exploring hybrid approaches to enhance its robustness and adaptability. Ultimately, this research showcases the feasibility and value of integrating machine learning into personalized medicine. By providing a reliable, interpretable, and efficient predictive framework, decision tree models pave the way for more informed clinical decision-making and better patient care.

## Statement of informed consent

As there was no intervention in the standard patient treatment process and only patient treatment data was utilized, informed consent was not obtained from the patients. Additionally, all patient information was securely archived at Shahid Madani Hospital in Tabriz. This study was approved by Tabriz University of Medical Sciences (MSc grant No. 59507).

## Ethics approval and consent to participate

At our study, there was no intervention in the standard patient treatment process and only patient treatment data was utilized, informed consent was not obtained from the patients. Additionally, all patient information was securely archived at Shahid Madani Hospital in Tabriz. Also, the method of study and patient data collection was conducted under the Helsinki criteria and the code of ethics of Tabriz University of Medical Sciences (MSc grant No. 59507). We confirm that all procedures and methods were performed in accordance with the guidelines and regulations of Tabriz University of Medical Sciences.

## Authors' contributions

A.M. and M. A.-H. Contributed to the study concept and design. M. A.-H. , H. Z. and A.Y contributed to data collection. A.K. Contributed to data analyses. All authors read and approved the final manuscript.

## Data availability statement

The datasets used and/or analyzed during the current study available from the corresponding author on reasonable request.

## Funding

This research received no external funding.

## Declaration of competing interest

The authors declare that they have no known competing financial interests or personal relationships that could have appeared to influence the work reported in this paper.

## Data Availability

Data will be made available on request.
